# Replacements of Rare Herbs and Simplifications of Traditional Chinese Medicine Formulae Based on Attribute Similarities and Pathway Enrichment Analysis

**DOI:** 10.1155/2013/136732

**Published:** 2013-02-21

**Authors:** Zhao Fang, Meixia Zhang, Zhenghui Yi, Chengping Wen, Min Qian, Tieliu Shi

**Affiliations:** ^1^Center for Bioinformatics and Computational Biology, Shanghai Key Laboratory of Regulatory Biology, Institute of Biomedical Sciences and School of Life Sciences, East China Normal University, Shanghai 200241, China; ^2^Department of Ophthalmology, West China Hospital, Sichuan University, Chengdu, Sichuan 610041, China; ^3^Schizophrenia Program, Shanghai Mental Health Center, School of Medicine, Shanghai Jiao Tong University, Shanghai 200030, China; ^4^TCM Clinical Basis Institute, Zhejiang University of Chinese Medicine, Hangzhou, Zhejiang 310053, China

## Abstract

A Traditional Chinese Medicine (TCM) formula is a collection of several herbs. TCM formulae have been used to treat various diseases for several thousand years. However, wide usage of TCM formulae has results in rapid decline of some rare herbs. So it is urgent to find common available replacements for those rare herbs with the similar effects. In addition, a formula can be simplified by reducing herbs with unchanged effects. Based on this consideration, we propose a method, called “formula pair,” to replace the rare herbs and simplify TCM formulae. We show its reasonableness from a perspective of pathway enrichment analysis. Both the replacements of rare herbs and simplifications of formulae provide new approaches for a new formula discovery. We demonstrate our approach by replacing a rare herb “*Forsythia suspensa*” in the formula “the seventh of Sang Ju Yin plus/minus herbs (SSJY)” with a common herb “Thunberg Fritillary Bulb” and simplifying two formulae, “the fifth of Du Huo Ji Sheng Tang plus/minus herbs (FDHJST)” and “Fang Feng Tang” (FFT) to a new formula “Fang Feng Du Huo Tang” (FFDHT).

## 1. Introduction

Traditional Chinese Medicine is an ancient system used in disease treatments for several thousand years already [1, 2]. Currently, TCM is popular only in Asia, but also in United States, Europe, and other countries as a complementary or alternative medicine [3, 4]. Nearly 100,000 TCM formulae have been recovered [5, 6], each of which normally contains several herbs. Here, “herbs” refer to not only plants, but also animals and minerals with effects of treatments [7].

An herb normally has five attributes: they have nature, taste, channel tropism, functions, and indications [8–10]. Nature refers to a basic characteristic based on the patient's reaction to an herb, and it has four properties: cold, cool, warm, and hot; taste indicates the actions an herb has on human body and it includes five characters: spicy, sour, sweet, bitter, and salty; channel tropism denotes an herb's selective therapeutic effects on a certain part of the body, and it includes twelve different organs: heart, liver, spleen, lung, kidney, stomach, large intestine, small intestine, bladder, gallbladder, pericardium, three warmers; functions signify the mechanisms of an herb's therapeutic effects. For example, a common herb and “Chinese angelica,” has the functions of “harmonizing the blood,” “regulating menses,” and “moistening intestines”; indications describe the diseases or symptoms treated by an herb. Several herbs compose a formula in the order of Master, Adviser, Soldier, and Guild [11], while a later order indicates less importance [12].

A formula usually contains many active compounds. These compounds target many molecules in the cell and work together to increase therapeutic efficacy and reduce adverse effects [6, 11]. Although great efforts have been made, the mechanisms of most formulae are still unknown [6, 13]. Recently, systems biology approaches [14] and TCM informatics [15] have been applied to explore the mechanisms for different formulae, but with a little significant progress. In this study, we explore the pharmacological effects of formulae from a perspective of pathway enrichment analysis. 

The availability of some herbs has a rapid decline with their wide usage [16]; for example “Shi-He Ming Yan Wan” [17], a powerful formula, has been used to treat many different diseases. It has been believed that the treatment power of the formula partially comes from a rare herb “rhinoceros horn,” which is widely used in many TCM formulae. Due to poaching and illegal trade, the number of black rhinos has shrunk by 96% since 1970 in Africa. The same situation has happened for “Pholidota,” “*Forsythia suspensce*,” “*Cordyceps sinensis*,” and so forth. So to continually use those formulae to treat the diseases, it is urgent to find the common available replacements for those rare herbs with the same effects [18].

Herb switches and simplification in a formula have been tested. For example, “Liu-wei-di-huang” (LWDH) [12], a famous formula with six herbs, was simplified from “Ba-wei-di-huang” (BWDH) by discarding two herbs based on herbs' attributes. In our study, we adopted the similar concept and simplified TCM formula with a “formula pair” method based on large-scale computation. We then showed the reasonableness of our approach with a perspective of pathway enrichment analysis, same as to the replacements of rare herbs.

## 2. Materials and Methods

### 2.1. Data Sources

We collected 4,343 formulae and 6,171 herbs from SIRC/TCM database (http://www.tcm120.com/1w2k/q_pres.asp). If several formulae have a same name but different herbs, we took the classical one (e.g., formula A) as a leader and named others as “the *n*th of formula A plus/minus herbs” (*n* = 1, 2,3…). Disease ontology identities (DOIDs) of diseases treated by formulae were obtained from human disease ontology.

### 2.2. Score System of Combinational Degree (CD) for Formula Pairs

Combinational degree refers to the similarity between two formulae. The CD was calculated to evaluate combinational degree between two formulae based on the herbs they share and the weights of shared herbs. Assuming there are two formulae *F*_1_ and *F*_2_. *L*_1_ and *L*_2_ are the number of herbs in *F*_1_ and *F*_2_, respectively, *n* is the number of shared herbs, herb *i* indicates one of the shared herbs, and *m*_1*i*_ and *m*_2*i*_ signify the sequential numbers of herb *i*'s positions in *F*_1_ and *F*_2_. The weights of herb *i* in *F*_1_ and *F*_2_, named *W*_1*i*_ and *W*_2*i*_, are calculated as

(1) $$\begin{array}{c} W_{1i}=1+\frac{\left(1-m_{1i}\right)}{L_{1}},\;\;W_{2i}=1+\frac{\left(1-m_{2i}\right)}{L_{2}}. \end{array}$$

The CD of *F*_1_ and *F*_2_ is calculated as

(2) $$\begin{array}{c} {\text{CD}}_{\left(F1,\;\;F2\right)}=\sum_{i=1}^{n}\left(\frac{W_{1i}+W_{2i}}{2}\right). \end{array}$$

An example is shown in Table 1.

We calculated all formula pairs of 4,343 formulae. The generated values for those CDs are from 0 to 7.5; we then normalized them to [0, 1] for convenient purpose. Combinational degree was used as an evaluation index of the similarity between two formulae. For a pair of formulae with a CD value of 0, we considered these two formulae without combination, which also means that they have no shared herbs. CD ∈ (0, 0.3] indicates the two formulae as low combination, while CD ∈ (0.3,0.7] as middle combination, and CD ∈ (0.7,1] as high combination based on the number of shared herbs in two formulae and the position of each herb in the formulae.

### 2.3. Disease Similarity Calculation for Each Formula Pair

DOSim [19] is an R package used to calculate the similarity between diseases using DOID based on disease ontology. For 4,343 formulae-treated diseases, only l,769 formulae-treated diseases can be mapped to DOIDs. Assuming that *p* and *q* represent the number of diseases treated by formulae *F*_1_ and *F*_2_, respectively, DOSim will generate *p*∗*q* similarity values for this formula pair. Each similarity value can be denoted as *K*_*i*_(*i* = 1, 2,…, *p*∗*q*). The similarity of treated diseases between *F*_1_ and *F*_2_, named as Sim, can be calculated as follows:

(3) $$\begin{array}{c} {\text{Sim}}_{1,2}=\frac{\sum_{i=1}^{p*q}K_{i}}{p*q}. \end{array}$$

For a pair of formulae with a Sim ∈ [0,0.3], we consider that those diseases the two formulae treat have no similarity, and a Sim ∈ (0.3,0.7] indicates those diseases with significant similarity for a formula pair, a Sim ∈ (0.7,1] with high similarity.

### 2.4. Score System of Attribute Similarities (AS) for Herb Pairs

To evaluate whether the attributes of two herbs are similar or not, a sore system was constructed for the purpose. For the five attributes in a formula, we define each one with a weight of 1. Based on a previous study, the detailed algorithms are as follows.

(1) The four natures of an herb are represented as *X*_1_, *X*_2_, *X*_3_, and *X*_4_, respectively. Different herbs have different natures. If an herb has only one nature, we reward this nature a value of 1 and other natures as 0. If the nature of an herb is “ping,” which means neutral, a value of 0.25 will be assigned to each of the four natures. A slight nature, for example, “slight cold,” will get a value of 0.8; for a severe nature, such as “severe cold,” we assign it a value of 1.2. For two herbs *i* and *j*, the similarity of their natures, named *A*_*i*,*j*_, is calculated as

(4) $$\begin{array}{c} A_{i,j}=\frac{\sum_{k=1}^{4}\min\left(X_{ki},X_{kj}\right)}{\sum_{k=1}^{4}\max\left(X_{ki},X_{kj}\right)}. \end{array}$$

An example is shown in Table 2.

(2) The five tastes are represented as *Y*_1_, *Y*_2_,…, *Y*_5_, respectively. According to the similar principal, if an herb has several tastes, we reward each of those tastes a value of 1 and 0 to other unidentified tastes. For a slight taste, such as “slight sour,” a value of 0.8 will be assigned. If the taste of an herb is described as “tasteless,” the value of “sweet” will be added 0.5. If the taste of an herb is depicted as “pucker,” the value of “sour” will get 0.5 bonus. For the two herbs *i* and *j*, the similarity of tastes, named *B*_*i*,*j*_, is calculated as

(5) $$\begin{array}{c} B_{i,j}=\frac{\sum_{k=1}^{5}\min\left(Y_{ki},Y_{kj}\right)}{\sum_{k=1}^{5}\max\left(Y_{ki},Y_{kj}\right)}. \end{array}$$

(3) The twelve channel tropisms are represented as *Z*_1_, *Z*_2_,…, *Z*_12_, respectively. According to the similar principal, if an herb has several channel tropisms, we reward each of those channel tropisms a value of 1 and 0 to other unidentified channel tropisms. For the two herbs *i* and *j*, their similarity of tastes, named *C*_*i*,*j*_, is calculated as

(6) $$\begin{array}{c} C_{i,j}=\frac{\sum_{k=1}^{12}\min\left(Z_{ki},Z_{kj}\right)}{\sum_{k=1}^{12}\max\left(Z_{ki},Z_{kj}\right)}. \end{array}$$

(4) The numbers of functions for herbs *i* and *j* are named as FT_*i*_ and FT_*j*_. FT_*i*,*j*_ is the number of shared functions between two herbs. The similarity of the functions between two herbs, named *D*_*i*,*j*_, is calculated as

(7) $$\begin{array}{c} D_{i,j}=\frac{2{\text{FT}}_{i,j}}{{\text{FT}}_{i}+{\text{FT}}_{j}}. \end{array}$$

An example is shown in Table 3.

(5) The numbers of indications for herbs *i* and *j* are named as IC_*i*_ and IC_*j*_. IC_*i*,*j*_ is the number of shared indications between two herbs. The similarity of the indications between two herbs, named *E*_*i*,*j*_, is calculated as

(8) $$\begin{array}{c} E_{i,j}=\frac{2{\text{IC}}_{i,j}}{{\text{IC}}_{i}+{\text{IC}}_{j}}. \end{array}$$

So, attribute similarity (AS) of herb *i* and herb *j* is calculated as

(9) $$\begin{array}{c} {\text{AS}}_{i,j}=A_{i,j}+B_{i,j}+C_{i,j}+D_{i,j}+E_{i,j}. \end{array}$$

If herbs *i* and *j* have an AS value greater than 3, they are considered to have high attributes' similarity.

## 3. Results

It has been a long history to use herbs to treat diseases. Herbs usually are any part of plants or certain animals with their medicinal effects. Since rare herbs decline rapidly with their wide usage, they face great threat of extinction. To protect those invaluable plants or animals, it is urgent to find the replacement for the formulae with rare herbs. Meanwhile, formulae are not changeless and can be simplified for the cost efficiency and availability. However, the prerequisites for replacements of rare herbs and simplifications of existed formulae are that the new formulae should not change the medical effects compared to original ones. According to this concept, we designed related strategy and used it to computationally detect the possibility of the replacement for all of the formulae we collected.

### 3.1. Replacements of Rare Herbs

Combinational degree refers to the similarity of two formulae; the smaller the degree, the less similarity between two formulae. Accordingly, the formula pairs with CDs ∈ (0,0.3] were used for herb replacement. With this criterion, two formulae in the pair share few herbs, thus, avoiding rare herbs shared by the two formulae, since if a rare herb is the shared herb for a formula pair, the herb used to replace the rare herb may be the rare herb itself according to our method. Assuming herb *i* is a rare herb in formulae *F*_1_ and herb *j* is a common one in *F*_2_, to replace herb *i* with herb *j*, we built a strict model as follows:

1. Sim_1,2_ ∈ (0.7,1],
2. AS_*i*,*j*_ > 3,
3. min(*W*_1*i*_, *W*_2*j*_)/max(*W*_1*i*_, *W*_2*j*_) > 0.8.

The replacement of herb *i* with *j* should meet all of the three conditions above. Condition (1) guarantees that herbs *i* and *j* contribute highly similar effects. Condition (3) assures that herbs *i* and *j* have similar weights. Under such circumstance, when herb *j* replaces herb *i* in *F*_1_, the order of Master, Adviser, Soldier, and Guild in *F*_1_ can be kept, and the impact of the replacement will be minimized and can be ignored. 

*Forsythia suspensa* is a rare herb protected by law due to its severe decline. According to our model, we replaced it in the formula “the seventh of Sang Ju Yin plus/minus herbs” (SSJY) with a common herb “Thunberg Fritillary Bulb,” which is a component (herb) in another formula “Sang Ke Tang” (SKT). Both SSJY and SKT can treat chronic bronchitis. Herbs in SSJY and SKT are shown in Table 4.

SSJY and SKT, meet the requirements in the replacement model based on the following results:

1. CD_(SSJY,SKT)_ = 0.162;
2. Sim = 0.999;
3. in SSJY, *W*_Forsythia  suspensa_ = 0.75; in SKT, *W*_Thunberg  fritillary  bulb_ = 0.75; *W*_Forsythia  suspensa_/*W*_Thunberg  fritillary  bulb_ = 1;
4. AS_(Forsythia  suspensa,Thunberg  fritillary  bulb)_ = 3.44.

So, we replaced “*Forsythia suspensa*” in SSJY with “Thunberg Fritillary Bulb” in SKT. The replacement resulted in a new formula and we named it as “Sang Ju Zhe Bei Yin” (SJZBY, Table 4).

### 3.2. Formulae Simplifications

To simplify a formula, we also built a model. For a formula pair with CD ∈ (0.3,0.7] and Sim ∈ (0.7,1], both of them can treat the same or highly similar diseases, we believe that the high similarity of the two formulae is caused by the shared herbs and the rest herbs with AS > 3. Under such assumption, two formulae can be simplified to a new formula which only consists of shared herbs and herbs with high attributes' similarity. 

**Figure 1:**
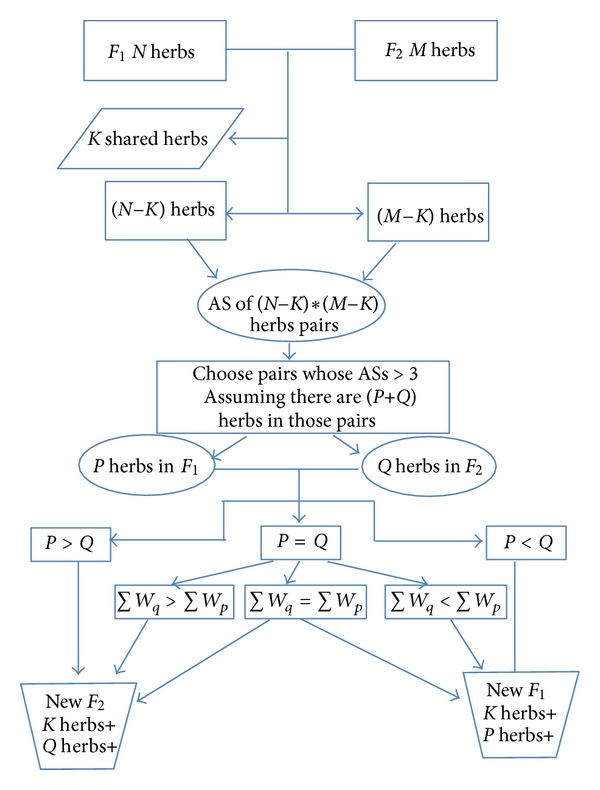
Workflow of simplification.

Assuming formulae *F*_1_ and *F*_2_ are a formula pair with CD ∈ (0.3,0.7] and Sim ∈ (0.7,1], *F*_1_ has *N* herbs, and *F*_2_ has *M* herbs; the number of shared herbs is *K*. For other (*N* − *K*) herbs in *F*_1_ and (*M* − *K*) herbs in *F*_2_, we calculate AS for each herb pair, and totally there are (*N* − *K*)∗(*M* − *K*) pairs. As a result, we then find out those pairs of herbs with their ASs > 3. If there are (*P* + *Q*) herbs in those formula pairs with ASs > 3 and there are *P* herbs in *F*_1_ and *Q* herbs in *F*_2_, the new formula can be generated as follows:

1. if (*P* > *Q*), new formula will consist of *K* shared herbs and *Q* herbs according to the principle of “least herbs.” This formula is named as “new *F*_2_”;
2. if (*P* < *Q*), new formula will consist of *K* shared herbs and *P* herbs. This formula is named as “new *F*_1_”;
3. if (*P* = *Q*), assuming ∑*W*_*p*_ is the total weights of *P* herbs in *F*_1_ and ∑*W*_*q*_ is the total weights of *Q* herbs in *F*_2_,
  1. if (∑*W*_*p*_ > ∑*W*_*q*_), new formula will be a “new *F*_1_” according to “higher weights, more importance” concept, the “new *F*_1_” formula will consist of *K* shared herbs plus *P* herbs;
  2. if (∑*W*_*p*_ < ∑*W*_*q*_), new formula will be a “new *F*_2_”;
  3. if (∑*W*_*p*_ = ∑*W*_*q*_), both “new *F*_1_” and “new *F*_2_” will be new formulae after discarding those unshared herbs with their ASs≦3. The workflow is shown in Figure 1.

For example, both formulae “the fifth of Du Huo Ji Sheng Tang plus/minus herbs” (FDHJST) and “Fang Feng Tang” (FFT) can treat rheumatoid arthritis (RA). FDHJST includes 15 herbs (Table 5) and FFT has 8 herbs (Table 5). They have 5 shared herbs: “Radix saposhnikoviae,” “Chinese angelica,” “Radix gentianae macrophyllae,” “Tuckahoe,” “Liquorice”. We calculated AS scores of herb-pairs for the rest unshared herbs and found that AS_(Root  of  doubleteeth  pubescent  angelica,  Notopterygium  root)_ was 3.44 and greater than 3. In FDHJST, *W*_Root  of  doubleteeth  pubescent  angelica_ = 1; in FFT, *W*_Notopterygium  root_ = 0.375. Since the weight of “root of doubleteeth pubescent angelica” in *F*_1_ is greater than the weight (0.375) of notopterygium root in *F*_2_, the new formula, named as “Fang Feng Du Huo Tang (FFDHT)”, will contain 5 shared herbs and “Root of doubleteeth pubescent angelica” (Table 5). 

### 3.3. Pathway Enrichment Analysis for SSJY, SKT, and SJZBY

To further verify that our formula replacement is relevant from biomedical view, we carried out pathway enrichment analysis with those target proteins for each formula. Protein targets of herbs in each formula were obtained from TCMID [10]. For SSJY and SKT, they have 21 shared compounds targeting 78 proteins. We used ClueGO [20] to conduct pathway enrichment analysis for those targets with 0.01 as the threshold of *P* value. The results showed that those targets were enriched in 39 pathways with *P* values less than 0.01. We ranked those pathway according to the *P* value of each enriched pathways in an ascending order and selected top 20 pathways (Table 6) for further mechanism analyses.

Among the top 20 pathways enriched by those 78 shared targets, we found that the pathway of cytokines and inflammatory response ranked 7th and the pathway of free radical-induced apoptosis ranked 11th. Both the two pathways were closely related to chronic bronchitis. 

The results showed that there were six targets enriched in the pathway of cytokines and inflammatory response. They are granulocyte-macrophage colony-stimulating factor (GM-CSF), tumor necrosis factor (TNF), interleukin-2 (IL-2), interleukin-4 (IL-4), interleukin-6 (IL-6), and interleukin-10 (IL-10). 

Inflammation has been proved to be a central factor to the development and progression of chronic bronchitis [21]. GM-CSF is capable of generating both granulocyte and macrophage colonies from precursor cells, and it has important functions in host responses to external stimuli and in inflammatory conditions [21]. Increased levels of GM-CSF have been found in the epithelium and bronchoalveolar lavage fluid from patients with chronic bronchitis [22]. For other targets, TNF shows statistically significant evidence of association with the susceptibility of chronic obstructive pulmonary disease such as chronic bronchitis [23]. IL-4, IL-6, and IL-10 are cytokines with anti-inflammatory effect [24]. The ingredients of both SSJY and SKT have significant impact on those targets, resulting in effective treatment to chronic bronchitis. Therefore, this pharmacological action of SSJY and SKT could be one of the main mechanisms for chronic bronchitis treatment.

Another pathway closely connected with chronic bronchitis is free radical-induced apoptosis. It has been reported that apoptosis of structural cells in the lung may contribute to the pathogenesis of chronic bronchitis [25, 26]. There is an increase in endothelial and epithelial apoptosis in the lungs of patients with chronic bronchitis which cannot be counterbalanced by an increase in proliferation, resulting in a destruction of lung tissue. Therefore, apoptosis has been proposed to be a therapeutic target [26]. Since targets of SSJY and SKT enrich in this pathway, the two formulae may inhibit the apoptosis in endothelial and epithelial cells by a multitarget effect. This pharmacological action of SSJY and SKT may be another main mechanism for chronic bronchitis treatment. Since those shared targets of SSJY and SKT are enriched in the two pathways, this could be the reason why both SSJY and SKT can treat chronic bronchitis. 

The new formula, SJZBY, also includes the same 78 targeted proteins. Therefore, it is reasonable to say that SJZBY should have the similar effect on the treatment of chronic bronchitis. Pathway enrichment analysis for shared targets of those formulae shows the reasonableness of this replacement.

### 3.4. Pathway Enrichment Analysis for FDHJST, FFT, and FFDHT

Pathway enrichment analysis was also applied to explore the potential mechanism for formula simplification. We collected the potential targets for formulae—FDHJST, FFT, and FFDHTl; they are 182, 133, and 95 proteins, respectively. The results show that targets of FDHJST, FFT, and FFDHT are enriched in 73, 64, and 53 pathways with *P* values less than 0.01, respectively. Same as the above mentioned method, we ranked those pathways according to the *P* value of each enriched pathway in an ascending order and selected top 20 pathways (Tables 7, 8, and 9) for further mechanism analyses.

In those top 20 pathways enriched by targets of FDHJST, we found that the pathway of cytokines and inflammatory response were closely connected with RA. The result showed that the *P* value of this pathway ranked second in the top 20 pathways. Further research showed that there were thirteen targets of FDHJST enriched in this pathway. They are GM-CSF, interferon beta (IFNB), interferon-gamma (IFNG), transforming growth factor beta-1 (TGFB1), TNF, interleukin-1 alpha (IL-1A), IL-2, IL-4, IL-5, IL-6, IL-8, IL-10, IL-13.

A previous report has confirmed that antagonism of GM-CSF represents a novel therapeutic approach for a variety of autoimmune-mediated inflammatory diseases, including RA [27]. IFNG can stimulate the production of chemokines and is a powerful activator of mononuclear phagocytes; IFNG has also been tried in immune-mediated diseases such as RA [28]. IFNB has shown antirheumatic potential [28]. TNF is proven to be expressed at high levels in rheumatoid joint tissue, where they contribute significantly to inflammation and articular destruction. TNF is the first cytokine to be fully validated as a therapeutic target for RA [29]. TGFB1 is highly expressed in joints in RA and is considered to be a regulator of anti-inflammation in RA [30]. Enhanced expression of TGFB1 protein has been detected in RA synovia and it may be related to the active pathological changes in RA synovia including synoviocyte hyperplasia, inflammatory cell infiltration, sublining angiogenesis, and granuloma formation [31].

For those interleukins, IL-6 and IL-8 can be found in RA pathway in KEGG pathway annotation [32]. IL-10, also known as human cytokine synthesis inhibitory factor, is an anti-inflammatory cytokine and has been proposed to treat RA in clinical practice because of its capacity to inhibit cellular immunity and deactivate macrophages [33]. In summary, considering the proteins of compounds from FDHJST target, the pathway enrichment analysis demonstrates the potential mechanisms of the formula on RA treatment.

Among those top 20 enriched pathways by targets of FFT, the pathway of cytokines and inflammatory response was closely connected with RA. The result showed that this pathway ranked third among the top 20 pathways according the *P* value. There were ten targets of FFT enriched in this pathway. They are GM-CSF, IFNB, TNF, IL-2, IL-4, IL-5, IL-6, IL-8, IL-10, IL-13, all of which are the same targets of FDHJST. This result indicates that the potential mechanism of FFT to treat RA is highly similar to that of FDHJST.

After simplification, the resulting new formula, FFDHT, was also enriched in the pathway of cytokines and inflammatory response with the *P* value ranked 11th among the top 20 pathways. Seven targets were enriched in this pathway. They are GM-CSF, TNF, IL-2, IL-4, IL-6, IL-8, IL-10, all of which are also the targets of FDHJST or FFT. Accordingly, it is reasonable to state that FFDHT inherits the pharmacological effects of original ones and has a highly potential effect the treatment of RA, which shows the reasonableness of this simplification.

## 4. Discussion

Many herbs used in Traditional Chinese Medicine are endangered, such as tiger bone used to treat rheumatism. Its widely usage results in the rapid decline of tigers with the poaching and illegal trade, which push tigers to extinction [17]. Another example is *Cordyceps sinensis*; the huge commercial demand of *Cordyceps sinensis* for its powerful tonic function has led to the excessive harvest and its dramatic decline [34]. Therefore, those invaluable herbs are hard to be obtained currently, and the practices in TCM face a great challenge to use those related formulae to treat the diseases. Finding the common replacements for those invaluable rare herbs is in high demand. In this study, we proposed a method to replace rare herbs with common available ones and showed its reasonableness from a perspective of pathway enrichment. The case study suggests the applicability for the replacement of rare herbs, which opens the gate for wide implementation in the field and could have the profound impact on this field.

Moreover, we also proposed a method to simplify formulae based on the similar rationale. A new formula can be formulated with “less herbs but same effect” concept to the original one. Pathway enrichment analysis also shows the reasonableness of the simplification. Our approaches provide an alternative way to reformulate those traditional prescriptions. 

Although herbs have been widely used for thousands of years, most of their targets are still unclear and the mechanisms underling their effects remain unknown. And that has strongly prevented the modernization of traditional Chinese Medicine. For example, in the method of score system of attributes' similarities for herb pairs, we found that “rhinoceros horn” and “Buffalo Horn” have high attributes' similarity (AS = 3.5). Replacement of “rhinoceros horn” with “Buffalo Horn” had been used in clinic, we would like to explore the rationale from a perspective of pathway enrichment. However, no targets of the two herbs have been inferred. And similar situation between “walnut kernel” and “*Cordyceps sinensis,*” we also found that “walnut kernel” has a potential to replace “*Cordyceps sinensis*” since the AS between them is 3.2. However, no targets have been identified yet. With the progress of pharmacological research on herbs, it can be anticipated that more and more targets will be identified; we believe that more replacements can be carried out based on our approaches and the potential mechanisms behind those replacements can be explored from a perspective of pathway enrichment.

In this work, both replacement of rare herbs and simplification of formulae were computationally tested; our approaches provide an alternative way for new TCM formulation and mechanism inference. To fully verify our method and test the effects of those new formulae, more preclinical experiments need to be conducted. By the combination of *in silico *and web lab approaches, we expect that the modernization of Traditional Chinese Medicine will be speeded up; thus, people will benefit from this progress.

## Figures and Tables

**Table 1:** Weight of each herb in *F*_1_ and *F*_2_.

Formula	herb 1	herb 2	herb 3	herb 4	herb 5	herb 6	herb 7
*F*_1_	1	0.8	0.6	0.4	0.2		
*F*_2_		1		0.75		0.5	0.25

*Assuming  *F*_1_ includes 5 herbs: herb 1, herb 2, herb 3, herb 4, and herb 5, while *F*_2_ includes 4 herbs: herb 2, herb 4, herb 6, and herb 7. Herb 2 and herb 4 are assumed as shared herbs of *F*_1_ and *F*_2_. Weight of each herb is shown in this table. So CD of *F*_1_ and *F*_2_ is calculated as CD_(*F*_1_,*F*_2_)_ = (0.8 + 1)/2 + (0.4 + 0.75)/2 = 1.475. Then CD_(*F*_1_,*F*_2_)_ was divided by 7.5 to 0.197 for normalization.

**Table 2:** Values of natures for herb *i* and herb *j*.

	Cold	Cool	Warm	Hot
Herb *i*	0.25	0.25	0.25	0.25
Herb *j*	0.8	0	0	0

*Assuming herb *i* has a nature of “ping” and herb *j* has a nature of “slight cold.” Values of natures are showed in this table. So *A*_*i*,*j*_ is calculated as *A*_*i*,*j*_ = 0.25/(0.8 + 0.25 + 0.25 + 0.25) ≈ 0.16.

**Table 3:** Functions and *D*_*i*,*j*_ of herb *i* and herb *j*.

Herb *i*	Function 1	Function 2	Function 3	Function 4	Function 5
Herb *j*	Function 2	Function 4	Function 6	Function 7	

*Assuming herb *i* has 5 functions and herb *j* has 4 functions. Function 2 and Function 4 are shared functions. So *D*_*i*,*j*_ is calculated as *D*_*i*,*j*_ = (2∗2)/(5 + 4) ≈ 0.44.

**Table 4:** Herbs in SSJY, SKT, and SJZBY.

Formula		Herbs		
SJYJJS	Mulberry leaf	Chrysanthemum	*Forsythia suspense *	Reed rhizome
	Common hogfennel root	Bitter apricot kernel	Platycodon root	Liquorice
SKT	Mulberry leaf	Fermented soybean	Thunberg fritillary bulb	Radix adenophorae
	White mulberry root-bark	Cape jasmine fruit	Bitter apricot kernel	Liquorice
SJZBY	Mulberry leaf	Chrysanthemum	Thunberg fritillary bulb	Reed rhizome
	Common hogfennel root	Bitter apricot kernel	Platycodon root	Liquorice

**Table 5:** Herbs in FDHJST, FFT, and FFDHT.

Formula	Herbs		
DHJSTJJF	Root of doubleteeth pubescent angelica	Mistletoe	Radix gentianae macrophyllae
	Radix saposhnikoviae	Manchurian wildginger	Chinese angelica
	Szechwan lovage rhizome	Chinese herbaceous peony	Drying rehmannia root
	Bark of eucommia	Radix achyranthis bidentatae	Ginseng root
	Tuckahoe	Cassia bark	Liquorice
FFT	Radix saposhnikoviae	Chinese angelica	Radix gentianae macrophyllae
	Cassia twig	Notopterygium root	Bitter apricot kernel
	Tuckahoe	Liquorice	
FFDHT	Radix saposhnikoviae	Chinese angelica	Radix gentianae macrophyllae
	Tuckahoe	Root of doubleteeth pubescent angelica	Liquorice

**Table 6:** Top 20 pathways enriched by shared targets of SSJY and SKT with *P* values less than 0.01.

Number	Pathway name	*P* value
1	Leishmaniasis	1.19*E* − 08
2	Pathways in cancer	1.83*E* − 08
3	Pertussis	2.41*E* − 07
4	HTLV-I infection	3.18*E* − 06
5	Chagas disease (American trypanosomiasis)	6.94*E* − 06
6	T-cell receptor signaling pathway	9.98*E* − 06
7	Cytokines and inflammatory response	1.81*E* − 05
8	Measles	8.82*E* − 05
9	Legionellosis	1.16*E* − 04
10	Amoebiasis	1.16*E* − 04
11	Free radical-induced apoptosis	1.31*E* − 04
12	Cytokine network	1.39*E* − 04
13	African trypanosomiasis	1.42*E* − 04
14	IL-5 signaling pathway	2.16*E* − 04
15	Colorectal cancer	2.66*E* − 04
16	Influence of Ras and Rho proteins on G1 to S transition	3.69*E* − 04
17	NF-*κ*B activation by nontypeable *Haemophilus influenzae *	3.69*E* − 04
18	Rheumatoid arthritis	3.86*E* − 04
19	Toll-like receptor signaling pathway	7.75*E* − 04
20	Signal transduction through IL1R	9.92*E* − 04

**Table 7:** Top 20 pathways enriched by targets of FDHJST with *P* values less than 0.01.

Number	Pathway name	*P* value
1	Pathways in cancer	4.17*E* − 21
2	Cytokines and inflammatory response	7.15*E* − 13
3	Colorectal cancer	5.71*E* − 12
4	Cytokine network	2.60*E* − 11
5	Malaria	4.64*E* − 11
6	Chagas disease (American trypanosomiasis)	4.63*E* − 10
7	Pancreatic cancer	7.47*E* − 10
8	Amoebiasis	9.38*E* − 10
9	Bladder cancer	1.27*E* − 09
10	Leishmaniasis	1.87*E* − 09
11	Pertussis	2.31*E* − 08
12	Tuberculosis	3.50*E* − 08
13	Legionellosis	5.52*E* − 08
14	Rheumatoid arthritis	7.19*E* − 08
15	Small cell lung cancer	1.53*E* − 07
16	Chronic myeloid leukemia	2.16*E* − 07
17	Prostate cancer	3.56*E* − 07
18	HTLV-I infection	3.64*E* − 07
19	Influenza A	6.90*E* − 07
20	African trypanosomiasis	1.26*E* − 06

**Table 8:** Top 20 pathways enriched by targets of FFT with *P* values less than 0.01.

Number	Pathway name	*P* value
1	Pathways in cancer	3.87*E* − 17
2	Colorectal cancer	1.17*E* − 11
3	Cytokines and inflammatory response	1.18*E* − 09
4	Cytokine network	2.52*E* − 09
5	Prostate cancer	3.27*E* − 09
6	Amoebiasis	5.10*E* − 09
7	Pancreatic cancer	2.22*E* − 08
8	Chagas disease (American trypanosomiasis)	3.30*E* − 08
9	Chronic myeloid leukemia	3.86*E* − 08
10	Pertussis	4.62*E* − 08
11	Leishmaniasis	4.62*E* − 08
12	Apoptotic signaling in response to DNA damage	1.85*E* − 07
13	Tuberculosis	2.32*E* − 07
14	Small cell lung cancer	2.39*E* − 07
15	Influence of Ras and Rho proteins on G1 to S Transition	6.39*E* − 07
16	p53 signaling pathway	6.79*E* − 07
17	Toxoplasmosis	1.23*E* − 06
18	Measles	1.51*E* − 06
19	Malaria	1.61*E* − 06
20	HTLV-I infection	2.85*E* − 06

**Table 9:** Pathways enriched by targets of FFDHT with *P* values less than 0.01.

Number	Pathway name	*P* value
1	Pathways in cancer	4.96*E* − 16
2	Colorectal cancer	4.32*E* − 11
3	Prostate cancer	3.23*E* − 10
4	p53 signaling pathway	5.15*E* − 08
5	Pertussis	1.66*E* − 07
6	Small cell lung cancer	6.70*E* − 07
7	Influence of Ras and Rho proteins on G1 to S transition	1.44*E* − 06
8	Endometrial cancer	1.49*E* − 06
9	Pancreatic cancer	1.49*E* − 06
10	Amyotrophic lateral sclerosis (ALS)	1.78*E* − 06
11	Cytokines and inflammatory response	2.68*E* − 06
12	Bladder cancer	4.68*E* − 06
13	Tuberculosis	4.79*E* − 06
14	Amoebiasis	9.81*E* − 06
15	Apoptosis	1.27*E* − 05
16	HTLV-I infection	1.35*E* − 05
17	Cytokine network	1.45*E* − 05
18	RB tumor suppressor/checkpoint signaling in response to DNA damage	1.61*E* − 05
19	Apoptotic signaling in response to DNA damage	2.01*E* − 05
20	Chronic myeloid leukemia	3.19*E* − 05
